# Badnaviruses of Sweet Potato: Symptomless Coinhabitants on a Global Scale

**DOI:** 10.3389/fpls.2020.00313

**Published:** 2020-03-31

**Authors:** Jan F. Kreuze, Ana Perez, Marco Galvez Gargurevich, Wilmer J. Cuellar

**Affiliations:** Virology laboratory, International Potato Center (CIP), Lima, Peru

**Keywords:** *Ipomoea batatas*, Caulimoviridae, episomal, siRNA, genome integration

## Abstract

Sweet potato is among the most important root crops worldwide, particularly in developing countries, and its production is affected severely by a variety of virus diseases. During the last decade, a number of new viruses have been discovered in sweet potatoes through next-generation sequencing studies. Among them are viruses belonging to the genus *Badnavirus* and collectively assigned to the species *sweet potato pakakuy virus* (SPPV). We determined the complete genome sequence of two SPPV isolates and show the ubiquitous presence of similar viruses in germplasm and field material from around the globe. We show that SPPV is not integrated into the sweet potato genome, occurs only at extremely low titers, and is efficiently transmitted through seeds and cuttings. They are unaffected by virus elimination therapy and do not induce discernible symptoms in sweet potatoes or indicator host plants. They show considerable variation in their nucleotide sequences and correspond to several genetic lineages. Studies of their interaction with the two most important sweet potato viruses showed only limited synergistic increase in the titers of one of two SPPV isolates. We contend that these viruses may pose little threat to sweet potato production and more likely represent a new type of persistent virus in sweet potato.

## Introduction

Sweet potato is one of the most important food crops worldwide and is also used for animal feed, as well as for processing. In developing countries, it often serves as a food security crop for subsistent farmers, able to yield even in circumstances where other crops fail. Currently orange-fleshed varieties are being promoted in sub-Saharan Africa to combat vitamin A deficiency due to their high content of pro-vitamin A. Being clonally propagated, sweet potatoes suffer from the accumulation of viral diseases over generations, leading to reduced yields. More than 30 viruses have been reported from sweet potato to date, with most belonging to the families Potyviridae, Geminiviridae, and Caulimoviridae (Clark et al., 2012). The most important disease of sweet potato is known as the sweet potato virus disease complex (SPVD). It is caused by the synergistic coinfection of sweet potato chlorotic stunt virus (SPCSV; genus *Crinivirus*, family Closteroviridae) and sweet potato feathery mottle virus (SPFMV; genus *Potyvirus*, family Potyviridae) and may be exacerbated by infection with additional viruses (Mukasa et al., 2006; Untiveros et al., 2007).

Some of the more recently discovered viruses in sweet potato are the sweet potato badnaviruses variants A and B (Kreuze et al., 2009), which have collectively been assigned to the species *sweet potato pakakuy virus* (SPPV, family Caulimoviridae, genus *Badnavirus*). Although SPPV has already been identified on all continents using small RNA or total RNA sequencing and polymerase chain reaction (PCR) (Mbanzibwa et al., 2011, 2014; Kashif et al., 2012; Mingot et al., 2016; Qin et al., 2016), little is still known about the biology of this group of viruses. Badnaviruses (Bhat et al., 2016) infect a broad range of important crops including monocots and dicots, although most species have limited host ranges. They often infect perennial crops, and symptoms induced by badnaviruses range from moderate to very mild, or absent. They are easily spread long distances through vegetative planting materials, although efficient seed transmission is also known for some species. Horizontal transmission has been reported by various mealybug or aphid species, depending on the virus species. Pararetroviruses, including badnaviruses, can be present as integrated sequences in the genomes of some host plants. Such sequences are termed endogenous pararetroviruses (EPRVs). Whereas such sequences are often fragmented and unable to reconstitute an infective viral genome, some EPRVs can be reactivated by stress conditions and form actively replicating viruses. This occurs, for example, with banana streak viruses in some banana accession (Chabannes and Iskra-Caruana, 2013). Integration takes place through illegitimate recombination and is not necessarily associated with infection by a replicating virus. Southern blot analysis, immune-capture reverse transcriptase (RT)–PCR, and rolling circle amplification (RCA) are some of the techniques that have been employed to distinguish EPRVs from episomal viruses.

The aim of our study was to investigate (i) whether SPPV sequences found in sweet potato represented genome integrated and/or episomal viral sequences, (ii) whether they could be transmitted to other plants horizontally through grafting or vertically through seed, (iii) whether they could be associated with any symptoms, (iv) how common and variable they are in sweet potato germplasm, and (v) if they showed any (synergistic) interaction with the two most common sweet potato viruses SPFMV and SPCSV.

## Results

### SPPV Viruses Are Highly Variable and Ubiquitous Among Sweet Potato Accessions

**FIGURE 1:**
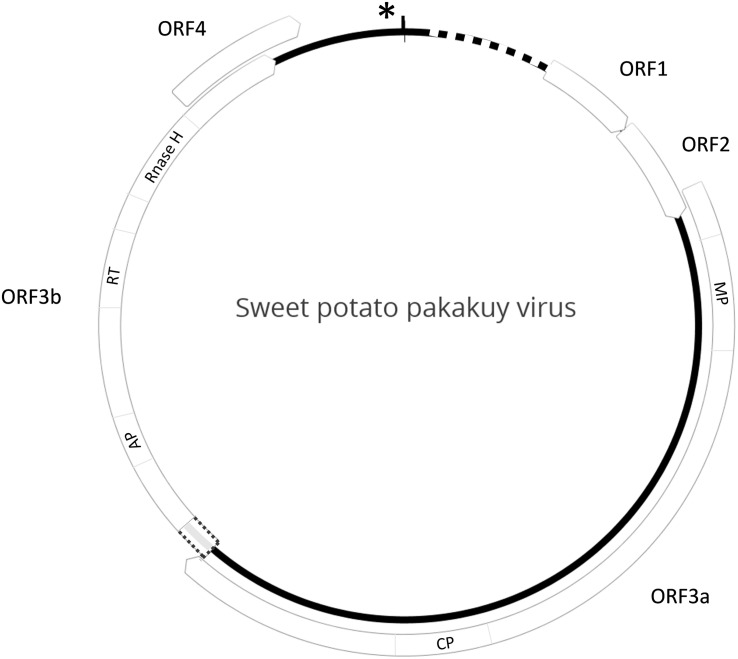
Genome structure of SPPV. Diagram depicting the genome structure of sweet potato pakakuy virus (SPPV). Circle indicates the genome with box arrows indicating the locations of predicted ORFs and numbered in order of occurrence. The dotted box between ORF3a and 3b indicates a region of uninterrupted ORF prior to the ORF 3b start codon that could serve as a potential read-through domain from ORF 3a. Protein domains encoded by ORFs are indicated inside the box arrows where known: MP, movement protein; CP, coat protein; AP, aspartyl protease; RT, reverse transcriptase; RNaseH, RNase H domain. Star and vertical black line indicate the location of the tRNA-met like region and short stem-loop structure, respectively, whereas the dotted line indicates location of a predicted leader sequence.

Entire genome sequences of SPPV variants A and B were completed and found to be 7,380 and 7,961 nt in length, respectively. Their genome structures were representative of typical badnaviruses, except that ORF3 was separated into two halves, which we designated ORF3a and ORF3b. ORF3a contains the movement and coat protein domains, and ORF3b includes the aspartyl protease, RT, and RNaseH (RH) domains (Figure 1). ORF3a and b are separated by a short non-coding region that is highly variable between the viruses and was sequenced several times from independently amplified and cloned PCR products to ensure accuracy. For both viruses, ORF3b is extended prior to the first methionine codon to overlap partially with ORF3a (12 and 21 nt, respectively, in SPPV-A and SPPV-B) and is found in a + 1 reading frame as compared to ORF3a. SPPV-A and SPPV-B share 79.5%-nt identity over the complete genome and shared the same tRNA-met–like region (TGG TAT CAG AGC GAG TAT) followed by a short stem-loop (GGC AGG CTA AGC CTA CC) and a putative leader sequence with extensive secondary structure (Figure 1).

To determine how common these badnaviruses were in sweet potato germplasm held at the International Potato Center (CIP), we screened a collection of 78 sweet potato genotypes from diverse geographic regions available in CIP’s germplasm collection with primers specific to SPPV-A and SPPV-B (Table 1) and found that many genotypes were infected by at least one of these viruses (Table 2).

**TABLE 1:** List of primers used in the detection for SPPV and qRT-PCR.

Target virus primers^a^		Sequence^b^ (5′–3′)	Size (bp)
SPPV A	SPBadna 2 5200-F	AATAATCCTCTCCTTCACTGGACAGAT	600
	SPBadna 1 5800-R	GATCCTCATGCTCTTCTTCAT	
	SPBadna2 3150 F	CAACTACACTGAACCATATGTCTCTC	400
	SPBadna1 3550R	AGTACCAAGGTCACCCGGCAC	
	SPBadna2 1750 F	TCGAGGAATGGTAGGAAGATTATC	1,400
	SPBadna2 3150 R	GAGAGACATATGGTTCAGTGTAGTTG	
SPPV B	SPBadna 1 5200-F	AGGTGGAATGCACGCTCAGGA	600
	SPBadna 2 5800-R	TTAAATGTTGCTCATGGTCCTCTTCTG	400
	SPBadna1 3150F	CTACAACTCTCAACCATATGTCCCTC	1,050
	SPBadna2 3550 R	TGGAACCAAGATCAAGGAAGAA	500
	SPBadna2 3550F	TGGAACCAAGATCAAGGAAGAA	600
	SPBadna 4600R	TCCTGATGCCGATGATATGATCTG	
	SPBadna 2700f	GAGAAGTTCAACGACAAGAAAGGAG	
	SPBadna2 3150r	GAGAGACATATGGTTCAGTGTAGTTG	
	SPBadnaB 5704f	AGGTGGAATGCACGCTCAGGATTA	
	SPBadnaB 6262r	AATGTTGCTCATGGTCCTCTTCTG	
SPPV RT	Pakakuy RT-F Pakakuy RT-R	CARGAYCCICCICTGAAGCATGT	700
		CCTARCCAMGATCTTARCCCTTTCTT	
SPPV RH	Pakakuy RT-F Pakakuy RH-R	CARGAYCCICCICTGAAGCATGT	900
		CCCAWCCWTCCATRCANCCRTC	
Begomovirus	SPG1 F^c^	CCCCKGTGCGWRAATCCAT	920
	SPG2 F^c^	ATCCVAAYWTYCAGGGAGCTAA	
SPFMV	SPF-F^d^	GGATTAYGGTGTTGACGACACA	589
SPVG	SPG-F^d^	GTATGAAGACTCTCTGACAAATTTTG	1,191
SPVC	SPC-F^d^	GTGAGAAAYCTATGCGCTCTGTT	836
SPFCG2	SPFCG2R^d^	TCGGGACTGAARGAYACGAATTTAA	
SPPV-A	rt-badA-left*	CCAACCCTCCTATGCACCT	61
	rt-badA-right*	AGTCGGGGGTCCACTTATCT	
SPPV-B	rt-badB-left*	TCGGCAGTAACAGACTACTTGG	147
	rt-badB-right*	TCTGCTTATCATCTCCGTTGG	
Sweet potato *Actin*	rt-swt-actin-left*	TTCTCCTTTCTAACACTCCTCAG	60
	rt-swt-actin-right*	CGCCTCGCTCTCTCTAGATCC	
Cox gene	COX-F*	CGTCGCATTCCAGATTATCCA	57
	COX-R*	AACTACGGATATATAAGAGCCAAAACTG	

^*a*^F, forward sense primer; R, reverse antisense primer. ^*b*^Y: C or T; M: A or C; R: A or G;W: A or T; I: inosine. ^*c*^Primers reported by Li et al. (2004). ^*d*^Primers reported by Li et al. (2012). *Primers used for qRT-PC.

**TABLE 2:** List of accessions used in this study and results of PCR tests for specific regions of SPPV-A and SPPV-B and degenerate primers.

Accession number	Accession name	Origin	SPPV A			SPPV B				RT	RH	Control
			1	2	3	1	2	3	4			
400171	Amarillo	Bolivia		−	−	−	−			+	+	+
440034*	Mohc	Burundi	−	−	−	−	+	−	−			+
440294*	Totokoitu	Cook Islands	−	−	−	+	−	+	−			+
400450	Bogotana	Colombia	−	+	−	−	−	−	−	+	+	+
440205	Unknown	China	−	+	−	−	−	−	−	+	+	+
440024	Yanshu 1	China	−	−	+	+	+	+	+		+	+
400584*	Bonito	Cuba	+	+	−	−	−	−	−			+
400632*	Santiaguero	Cuba	+	−	+	+	+	+	−			+
400822	Canabacoa	Dominican Republic	−	−	−	−	−	−	−	+	+	+
400830	Hoja de Panamacho	Dominican Republic	−	−	−	−	−	−	−	+	+	+
400028*	Violaceo (Puerto Rico)	Dominican Republic	−	−	+	+	+	+	−			+
400034	Unknown	Dominican Republic		−	−	−	−			+	+	+
400002*	Morado	Ecuador	−	−	+	+	+	−	−			+
441729*	Blanco Ecuatoriano	Ecuador	−	+	+	+	+	+	−			+
401111	Camote Morado	Guatemala	−	−	−	−	−	−	−	−	+	+
401104	Camote Naranja	Guatemala	−	−	−	−	−	−	−	+	+	+
401055*	Camote Blanco	Guatemala		−	−	+	+					+
400023*	Colombia. 633	Guatemala	−	−	−	−	−	−	−			+
401152	Rojo	Honduras		−	−	−	−			+	+	+
401154	Unknown	Honduras	−	−	−	−		−	−	+	+	+
440283*	BIS 50	Indonesia	−	+	+	+	+	−	+			+
440214*	BIS 99	Indonesia	−	−	−	−	−	−	−			+
401169	Herbie	Jamaica	−	−	−	−		−	−	+	+	+
440116	Gokoku-imo	Japan	−	−	+	+	+	−	−	+		+
440295	Seranggoon	Malaysia		−	+	+	+			+	+	+
401212	Regional de Tehuantepec	Mexico	−	−	−	−	−	−	−	+	+	+
401215*	Coleccion Tierra Blanca	Mexico	−	−	−	−	+	−	−			+
441724*	Cuitzeo	Mexico	−	−	+	+	+	+	−			+
400010*	226	Mexico	−	−	−	−		−	−			+
440398*	500 (PI 308201)	New Zealand	+	−	−	−	+	−	−			+
401223	Cubano	Nicaragua	−	−	−	−		−	−	+	+	+
401224	Camote	Nicaragua	−	−	−	−		−	−	+	+	+
401225	Camote	Nicaragua	−	−	−	−	−	−	−	+	+	+
401226	C-15	Nicaragua	−	−	+	+	+	+	−		+	+
401227*	CEMSA-74-228	Nicaragua	−	−	+	+	+	+	+			+
401228*	Batata Morada	Nicaragua	−	+	+	+		+	−			+
401243	Amarilla	Panama	−	−	−	−	−	−	−	+	+	+
401248	Amarillo	Panama	−	−	−	−	+	−	+			+
401253	Camote	Panama	−	−	+	+		+	−		+	+
440129	Ma’alua	Papua New Guinea	−	−	+	+	+	−	−	+	+	+
440305	Tawa-1	Papua New Guinea	−		+	+	+	−	−	+	+	+
400030*	Brasilera	Paraguay	−	−	−	−	−	−	−			+
400032*	Yety Aba	Paraguay		−	+	−	−					+
420509	Camote Amarillo	Peru	−	−	−	+	+	−	−	+	+	+
420617*	Chilpo Blanco	Peru	−	−	−	+	+	−	−			+
420065	Huachano	Peru	+	+	+	+	+	+	+	+		+
440160	Philippine	Philippines	−	−	+	+	+	+	−	+	+	+
440052	Margarita (SPV 70)	Puerto Rico	−	−	+	+	+	+	−	+	+	+
440163*	MUgandae	Rwanda	−	−	−	+	+	−	−			+
440202*	Ngiriare (ACC 275)	SLB	−	+	+	+	+	+	+			+
440360*	Iqui (ACC 78)	SLB		−	−	+	−					+
441169	So 272	SLB	−	−	−	−	−	−	−	+	+	+
400025*	LOVERs NAME	St vIncent and Grenadines	−	−	−	−	+	−	−			+
440197	Man Sai Daeng	Thailand	−	−	−	−	−	−	−	+	+	+
440343*	Unknown	Thailand	−	−	−	+	+	+	−			+
440348*	Kao	Thailand			+	+	+					+
440274	Kaloti	Tonga	−	−	−	+	+	−	−	+	+	+
440277*	Siale	Tonga	−	−	−	+	+	+	+			+
440012*	W-217	United States	−	−	−	−	−	−	−			+
440011	W-216	United States	−	−	+	+	+	−	−	+	+	+
440132	Beauregard	United States								+	+	
401403	Morado	Venezuela	−	−	−	−		−	−	+	+	+
401396*	Unknown	Venezuela		−	−	−	+	−	−			+
441726*	Tacarigua	Venezuela	+	−	−	−	−	−	−			+
400020*	No 2743	Venezuela		−	−	+	+	−	−			+
440267*	Hung Loc 4	Vietnam	−	−	−	−	−	−	−			+
440145	CAMEROUN 1112	Cameroun	+	+	+	+	+	+	−	+	+	+
440146	CAMEROUN 1592	Cameroun	−	+	+	+	+	+	+		+	+
440143*	CMR 048	Cameroun	−	−	−	−	+	−	−			+
440144	CMR 502	Cameroun	+	−	−	−	−	−	−	+	+	+
440390*	TIS 87/0087	Nigeria	−	−	+	+	+	+	−			+
440165	Kawogo	Uganda	−	−	+	+	+	+	−		+	+
440166	Tanzania	Uganda	−	−	+	+	+	+	−	+	+	+
Field	Bitambi	Uganda								+	+	
Field	KSR675 NORAII	Uganda								+	+	
Field	KSR675 Kameri Ikumi	Uganda								+	+	
Field	Marooko	Uganda								+	+	
Field	Carrot C	Tanzania								+	+	
460397*	*Ipomoea tiliacea*	Nicaragua	−	−	+	+	+	+	−			+
107665.9	*Ipomoea trifida*	Peru								+	+	
107665.19	*I. trifida*	Peru								+	+	

“−” and “ + ” indicate PCR test with primers of the corresponding column was negative or positive, respectively. If nothing is indicated in the cell, the PCR test was not performed for the corresponding primers and sample. *These accessions were not tested with degenerate primers.

**FIGURE 2:**
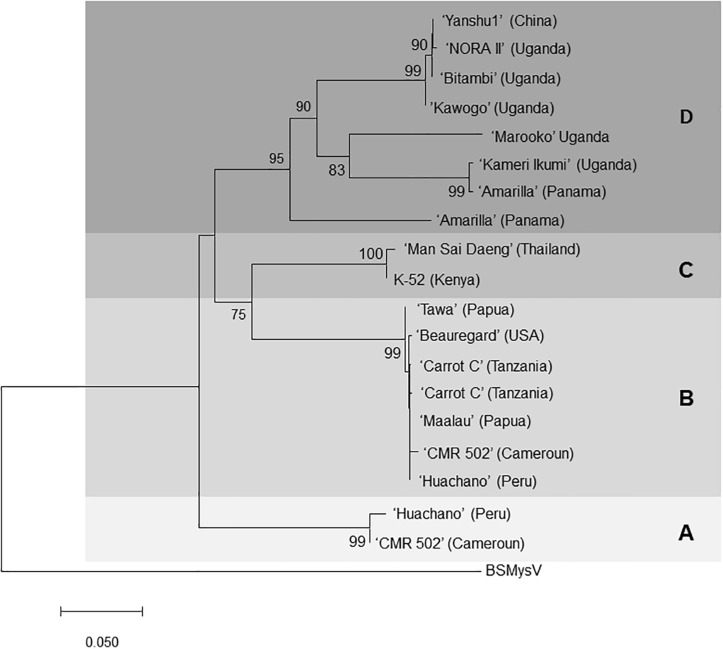
Phylogenetic tree of SPPV sequences covering the reverse transcriptase and Rnase H domains amplified from sweet potato accessions from around the world. The evolutionary history was inferred by using the minimum evolution method, and the evolutionary distances were computed using the maximum composite likelihood method and are in the units of the number of base substitutions per site. The optimal tree with the sum of branch length = 1.15686856 is shown. and is drawn to scale, with branch lengths in the same units as those of the evolutionary distances used to infer the phylogenetic tree. The percentage of trees in which the associated taxa clustered together is shown next to the branches based on 500 bootstrap replications when larger than 70%. The ME tree was searched using the close-neighbor-interchange algorithm at a search level of 1. The neighbor-joining algorithm was used to generate the initial tree. The analysis involved 20-nucleotide sequences. All ambiguous positions were removed for each sequence pair. There were a total of 828-nt positions in the final data set. Evolutionary analyses were conducted in MEGA7 (Lodhi et al., 1994). Isolates are indicated by the name of the variety from which they were amplified, and the origin of the variety is provided in brackets for each of them. BSMYV (banana streak MY virus) was used as an outgroup for phylogenetic tree construction. Four phylogenetic groupings **A, B, C,** and **D** are highlighted in different shades of gray.

Subsequent siRNA high-throughput sequencing and assembly of bulked RNA extracts, which included samples recently received from Africa, produced additional contigs corresponding to badnaviruses. Some of these contigs were clearly distinct from SPPV-A and SPPV-B (Supplementary Data S1). Based on alignments of the RT and RH domains of the various sequences obtained, degenerate primers (Table 1) were designed and used to amplify the corresponding region from a subset of the 78 sweet potato accession but also including five samples from African germplasm (Table 2). Analysis of alignments of nt or aa sequences of the RT or RT-RH domains resulted in a phylogenetic tree with three distinct and strongly supported clades, irrespective of the evolutionary inference method used, and a third more variable group, with less consistent support between phylogenetic inference method and/or nt substitution model applied (not shown). Two of the clades corresponded to SPPV-A and SPPV-B, whereas the new clades were designated C and D (Figure 2). Whereas clades A to C were rather homogeneous, with mean within-group nt variation of 1.1% to 2.2%, clade D was more variable with a mean variability of 10.5% and identifiable subgroupings. Inclusion of additional sequences corresponding to SPPV from the GenBank did not affect the grouping into these clades (data not shown).

### SPPV Can Be Graft Transmitted to Indicator Plants

Grafting experiments from sweet potato (cv. “Huachano” infected with SPPV-A and SPPV-B) to sweet potato (cvs. “Man Sai Deng” infected with SPPV-C and “Amarilla” infected with SPPV-D, but not with SPPV-A or SPPV-B) or to *Ipomoea setosa* followed by PCR of the grafted plants resulted in positive reactions in some cases (treatments 4, 5, 9, and 12 in Table 3) indicating that SPPVs could be transmitted through this means. Transmission was not with 100% efficiency, because in most cases only SPPV-B was transmitted (treatments 4, 5, and 9 in Table 3), whereas neither virus was transmitted to either sweet potato cultivar when the source plant “Huachano” was also infected by SPFMV and SPCSV (SPVD; treatments 10 and 13 in Table 3). To ensure that the virus detected in the graft inoculated *I. setosa* did not represent passively carried particles, the PCR-positive *I. setosa* plants were used to graft inoculate a second *I. setosa*, which subsequently became PCR positive upon testing, except when the *I. setosa* was also infected by SPFMV and SPCSV (treatments 6 and 7, respectively, in Table 3). Visible symptoms were only observed in plants infected with SPFMV + SPCSV (SPVD). Cloning and sequencing of the PCR fragments from the serially inoculated *I. setosa* plants confirmed they were identical to the sequence in the originally grafted plant in all cases.

**TABLE 3:** Results of graft transmission experiments.

Treatment	Plants	PCR results*		
		SPPV-A	SPPV-B	RT
1	Huachano^1^	1/1	1/1	1/1
2	Huachano (SPVD)^1^	1/1	1/1	1/1
3	*I. setosa*^2^	0/2	0/2	0/2
4	*I. setosa* + 1^3^	0/2	2/2	2/2
5	*I. setosa* + 2	0/2	2/2	2/2
6	*I. setosa* + 4	ND	ND	2/2
7	*I. setosa* + 5	ND	ND	0/2
8	Man Sai Deng^2^	0/2	0/2	2/2
9	Man Sai Deng + 1	0/2	2/2	2/2
10	Man Sai Deng + 2	0/2	0/2	2/2
11	Amarilla^2^	0/2	0/2	2/2
12	Amarilla + 1	2/2	2/2	2/2
13	Amarilla + 2	0/2	0/2	2/2

*Number of PCR-positive plants/number of plants tested with primers for SPPV-A, SPPV-B, or degenerate primers for *Badnavirus* reverse transcriptase (RT). ND, not done. ^1^Source plants; Huachano is naturally infected with SPPV-A and SPPV-B. We used another Huachano, which we additionally infected with SPFMV and SPCSV (SPVD). These plants were used to graft inoculate virus-free *I. setosa* plants in treatments 4 and 5, and sweet potato genotypes “Man Sai Deng” in treatments 9 and 10, and “Amarilla” in treatments 12 and 13. *I. setosa* plants from treatment 4 and 5 were further grafted to new virus-free *I. setosa* plants in treatments 6 and 7 as described in *Graft Transmissions*. ^2^Test plants before grafting. ^3^Test plant 25 days after graft inoculation with indicated source plant (treatment number) after the “+.”

### SPPVs Are Seed Transmitted in Sweet Potato

A previously generated *in vitro* germinated population from a cross between the cultivars Beauregard and Tanzania (Kyndt et al., 2015), which were both infected by SPPV (Tables 2, 4), was tested by PCR for the presence of SPPV in the established *in vitro* plants, and all 76 tested plants were found to be positive. PCR fragments were sequenced from “Beauregard” (the mother), as well as three progenies, and found to be greater than 99% identical to each other. Sequence similarity and phylogeny indicated this virus corresponded to SPPV-B (data not shown). In contrast, all seedlings tested negative by PCR for begomoviruses (using generic primers), which both parents were also infected with, and also were PCR negative for SPFMV, sweet potato virus G (SPVG), and sweet potato virus C (SPVC), which were infecting the parent “Beauregard” (Table 4). Thus, SPPV was transmitted to seed at very high efficiency.

**TABLE 4:** Results of PCR testing of *in vitro* germinated seedlings and their parents, Beauregard (B) and Tanzania (T).

Virus identified	SPPV	Begomovirus^#^	SPVG	SPVC	SPFMV
Beauregard	1/1*	1/1	1/1	1/1	1/1
Tanzania	1/1	1/1	0/1	0/1	0/1
B × T seedlings	76/76	0/76	0/76	0/76	0/76

*Number of PCR positive plants/number of plants tested. ^#^Generic primers were used and fragments were not sequenced to determine which species of begomovirus was present.

### Viral Titers of SPPV Are Less Than One Copy per Cell

**FIGURE 3:**
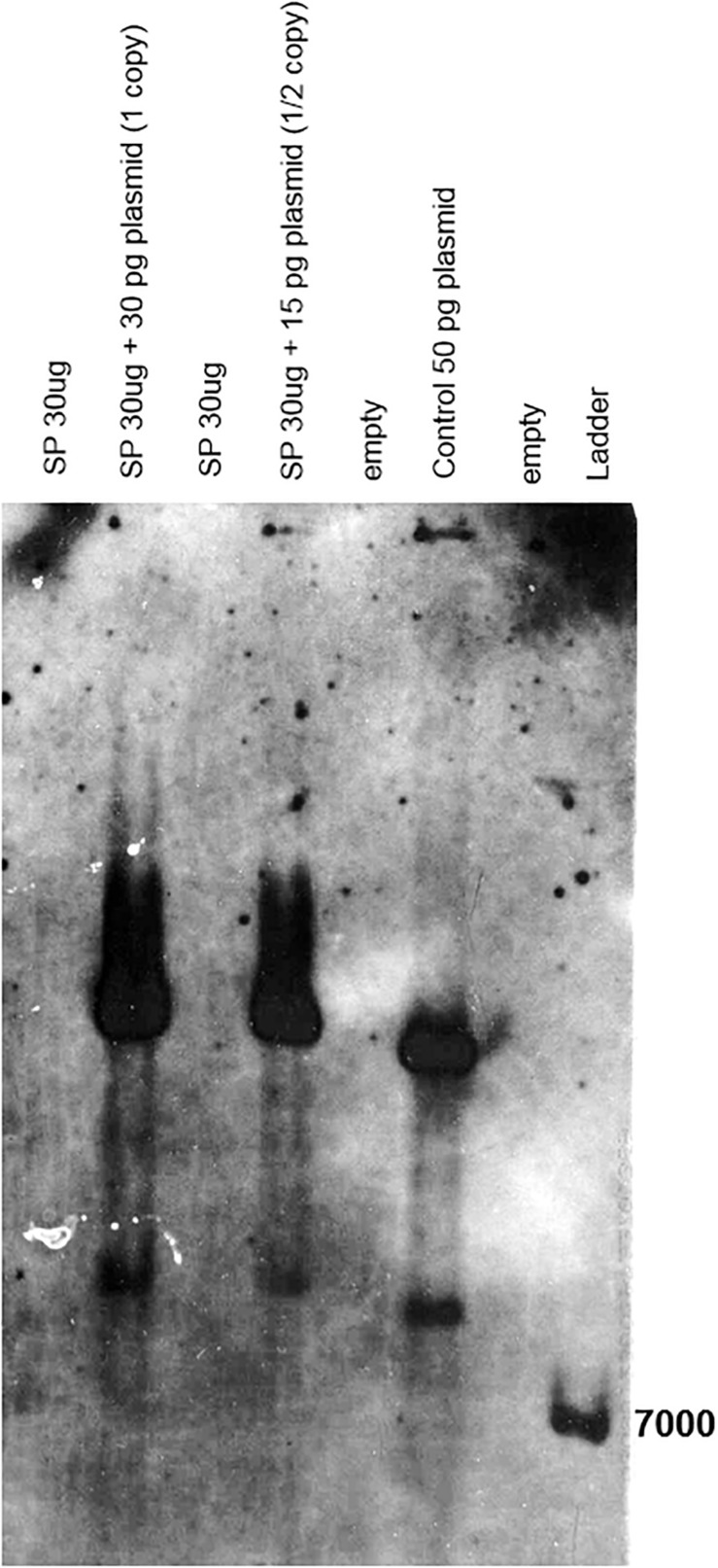
Southern blot of “Huachano” DNA linearized with *Pst*I and hybridized with a probe corresponding to SPPV-B. From left to right, the first and third lanes contain 30 μg of sweet potato (SP) DNA, and the second and fourth lanes contain 30 μg of SP DNA spiked with 30 and 15 pg of plasmid (containing SPPV-B DNA fragment corresponding to the probe), respectively, corresponding to 1 or one-half a copy per sweet potato genome equivalent; the fifth and seventh lanes are empty, whereas the sixth lane contains 50 pg of SPPV-B plasmid DNA, and the last lane, a DNA ladder.

Southern or dot blot experiments using SPPV-A– or SPPV-B–specific chemiluminiscent or radioactive probes consistently failed to detect either virus in several sweet potato accessions tested irrespective whether the plant was also infected by SPCSV, SPFMV, or both viruses (Figure 3 and data not shown). In contrast, these plants tested positive for SPPV by PCR (Table 2). On the other hand, sweet potato DNA spiked with plasmid DNA containing the SPPV-A or SPPV-B probe fragments at a concentration corresponding to one or half a copy per sweet potato genome were readily detected in Southern blot (Figure 3), indicating that the titers of these viruses must be well below these concentrations. This result implies these viruses are not integrated into the genome, as they should be present in at least one copy per cell, which could readily be detected in our assay.

### SPPV Titers Are Extremely Low and Are Only Minimally Affected by Coinfection of SPCSV and SPFMV, Whereas Corresponding siRNA Change Their Size Distribution and Are More Abundant in SPVD Affected Plants

**FIGURE 4:**
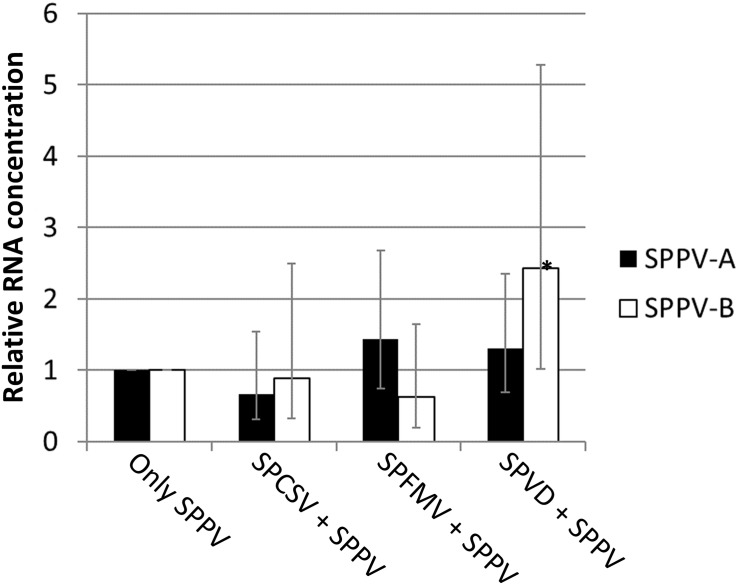
Relative expression for *Badnavirus* SPPV A and B. Bar graph depicting the expression of SPPV-A and SPPV-B in leaves in coinfection with SPFMV, SPCSV, or both viruses (SPVD) relative to plants infected only with SPPV (only SPPV). Error bars indicate standard error of relative expression. *Significantly upregulated as compared to plants infected only by SPPV (only SPPV; *p* = 0.001).

**FIGURE 5:**
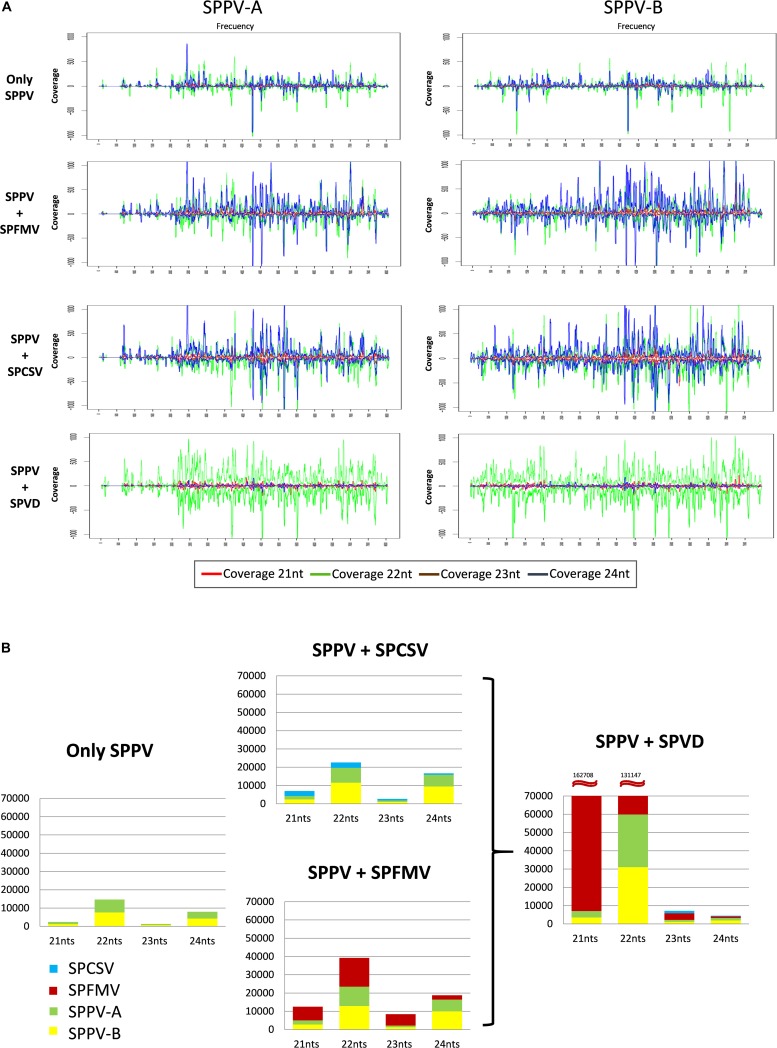
Size and distribution and quantities of siRNAs targeting SPPV in sweet potato plants coinfected with different viruses. **(A)** Graphics show the normalized distribution (per million siRNA reads sequenced) of siRNA covering the genomes of SPPV-A (left) and -B (right) in plants infected only by SPPV, or coinfected with SPFMV, SPCSV or SPFMV, and SPCSV (SPVD). The horizontal axis indicates the nucleotide position on the virus genome, whereas the vertical axis indicates the coverage of each nt position by siRNA sequences in sense (positive values) and antisense (negative values) orientation. Lines in red, green, brown, and blue represent 21-, 22-, 23-, and 24-nt siRNAs, respectively. **(B)** Bar graphics showing the normalized (per million siRNA reads sequenced) quantity (vertical axis) and size (horizontal axis) of virus-specific siRNAs in plants coinfected with different viruses. Green, yellow, red, and blue sections in the bars correspond to SPPV-A, SPPV-B, SPFMV, and SPCSV, respectively.

Because SPPV was below the detection limit of the Southern blot or dot blot methods, a quantitative real-time PCR assay was used to evaluate the distribution of virus titers in different leaves of sweet potato cv. Huachano. Results revealed quantitative RT-PCR (qRT-PCR) C(t) values averaging approximately six cycles below those of the reference gene *actin*, indicating extremely low concentrations in the extracted leaves (i.e., ∼1% compared to actin). In contrast, when comparing relative RNA levels of plants infected only with SPPV to those infected with SPFMV, SPCSV, or both viruses, a significant increase of approximately 2.5-fold (*p* = 0.001) could be identified only for SPPV-B in plants infected with both SPCSV and SPFMV (Figure 4). Mapping of siRNA sequences indicated that this correlated with increased siRNA production corresponding to SPPV-B viruses in plants infected by SPFMV and SPCSV as compared to other plants. This effect was observed mainly in siRNAs of 22-nt size, whereas 24-nt siRNAs were strongly reduced. The same effect could be appreciated also in SPPV-A (Figure 5).

## Discussion

Badnaviruses in sweet potato remain somewhat enigmatic. SPPV was initially identified through siRNA sequencing from apparently healthy plants thought to be virus-free (Kreuze et al., 2009) and has since then been identified in several next-generation sequencing (Kashif et al., 2012; Mbanzibwa et al., 2014; Mingot et al., 2016) studies and by PCR using specific primers (Mbanzibwa et al., 2011; Qin et al., 2016). Indeed, in this study, we found that every sweet potato plant tested turned out PCR positive for SPPV when degenerate primers were employed (Table 2). However, results were not always consistent over time in all plants; a plant could test positive for a leaf sample at one time and negative at others (data not shown), suggesting low and unequally distributed concentrations in the plant. Nevertheless, because some badnaviruses are known to exist as EPRVs, and EPRVs are also targeted by siRNAs through RNA silencing (Chabannes and Iskra-Caruana, 2013), it was important to confirm that we were not detecting integrated sequences. Our Southern blot experiments in Huachano (Figure 3) show that SPPV is not integrated in the genome of at least that cultivar and that SPPV concentrations are significantly below the equivalent of one copy per cell. This conclusion was supported by qRT-PCR results showing that the concentration of SPPV RNA was approximately a hundred-fold lower than that of the Actin reference gene (and ∼500 fold lower than COX reference). In addition, the size distribution of siRNAs mapped to the SPPV genomes showed that the relative abundance of 22-nt siRNAs was higher than that of 24-nt siRNAs (Figure 5B). EPRVs usually spawn predominantly 24-nt siRNA (Pooggin, 2018). The abundance of 22-nt siRNAs is consistent with episomal replication. On the other hand, we were unable to amplify SPPV by Phi29-mediated RCA from several cultivars (data not shown). However, this is perhaps unsurprising considering the limited sensitivity of RCA. Our pure control plasmids were only amplified by Phi29 from 1 ng or more of DNA (not shown), whereas our Southern blot and qRT-PCR experiments indicate that virus amounts were well below that. Sequence analysis of some of the amplified fragments, from plants originating from different parts of the world, showed considerable sequence variation between SPPV isolates found in different genotypes. It also showed that many genotypes were infected by more than one variant, similar to our findings in cv. Huachano (Figure 2). This result suggests that SPPV is an actively evolving virus.

Our virus transmission experiments showed that SPPV-A and SPPV-B could be transmitted by grafting to *I. setosa* and other sweet potato plants infected with SPPV-C or D. It is noteworthy that in most cases only SPPV-B was transmitted, whereas qRT-PCR results suggested that the initial titers of both viruses were very similar. Perhaps SPPV-B is more adept at establishing infections than SPPV-A in a competitive situation. The fact that SPPV-A and SPPV-B were found together and that SPPV-B could be transmitted to plants infected with SPPV-C or -D provided evidence that these viruses are not mutually exclusive. Coinfection of the source plant with SPFMV and SPCSV appeared to eliminate graft transmission of either virus to other sweet potato plants, and serial transmission to *I. setosa*. Sweet potato virus disease is a severe disease in sweet potatoes and sometimes lethal in *I. setosa*. It is conceivable that the stress caused by SPVD affects the formation of graft unions, which may impede efficient transmission of a virus present in low titers.

Qin et al. (2016) reported graft transmission of SPPV-A to *I. setosa* as determined by PCR, resulting in mosaic symptoms. However, it was not clear from that report whether other viruses were infecting the original sweet potato plants. It should also be noted that mosaic is not a typical symptom caused by badnaviruses. The findings of Qin et al. (2016) contrast with our findings, which could not identify symptoms in *I. setosa* after graft transmission. None of the plants tested in this study showed any clear virus symptoms (except when affected by SPVD). The extremely low virus titers determined by qRT-PCR in the accession Huachano suggest that only very few cells might be infected, and virus expression could be too low to induce any significant physiological changes in the plant that might manifest themselves in symptoms. However, without the availability of a plant lacking SPPV sequences, it will remain impossible to determine whether SPPV has any impact on sweet potato production. Our qRT-PCR and siRNA sequencing experiment in plants coinfected with SPFMV and SPCSV indicated only minor effects on SPPV titers, which were only significant in the case SPPV-B in dual infection with SPFMV and SPCSV. The modest twofold increase observed, however, seems unlikely to mediate much impact, particularly when considering the several hundreds of fold increase of different DNA and RNA viruses, caused by SPCSV coinfection (Karyeija et al., 2000; Mukasa et al., 2006; Cuellar et al., 2008, 2011, 2015). As had been previously observed (Kreuze et al., 2009), infection of both SPFMV and SPCSV had a marked effect on the amount and size of siRNAs targeting SPPV, but also infection by SPFMV and SPCSV alone affected siRNA amounts (but not size). These changes may be attributed to the silencing suppressors encoded by SPFMV and SPCSV (Kreuze et al., 2005; Cuellar et al., 2009). However, as evidenced from qRT-PCR experiments, coinfections with these viruses had minimal effect on SPPV titers. We noted that siRNA mapping to the viral genomes revealed a ∼300-bp region in the SPPV-A (but not SPPV-B) genome (Figure 5A), where no siRNAs mapped. This has not been observed for other *Badnavirus* sequenced by siRNA ((Pooggin, 2018) and references therein). This region was sequenced from several PCR fragments with ample overlap to flanking regions, which were targeted by siRNAs, and we currently have no explanation for this observation.

The genome organizations of the two SPPV isolates determined in this study are slightly different from most other badnaviruses in that ORF3 is divided into two (3a and 3b), a situation also found in jujube mosaic-associated virus (genus *Badnavirus*) (Du et al., 2017) and cassava vein mosaic virus (genus *Cavemovirus*) (De Kochko et al., 1998). Although ORF3b may be expressed from a separate mRNA, the possibility remains that it is expressed through + 1 ribosomal frameshifting as there is an overlap between the two ORFs when extending ORF3b 5’of its first potential initiation codon.

The “Huachano” plants originated from *in vitro* plants that had been submitted to thermotherapy and meristem tip culture for virus elimination. This suggests that SPPV is able to maintain itself in meristematic tissues. Indeed, attempts in other laboratories to eliminate viruses by thermotherapy and meristem excision failed to eliminate SPPV (Christopher Clark, personal communication). On the other hand, several accessions of wild sweet potato relatives, *Ipomoea tiliacea* and *Ipomoea trifida*, which are grown from seed, were also found to be positive suggesting that the virus could also be transmitted by seed. Seed transmission was confirmed to be highly efficient in sweet potato by testing *in vitro* germinated seedlings derived from a cross between “Beauregard” and “Tanzania,” whereas other viruses infecting either parent showed no evidence of seed transmission, as expected. Perhaps this is the principal mechanism by which SPPV has maintained and spread itself among sweet potatoes worldwide as vector transmission is unlikely at such low virus titers. Yet, the sequence variation found between different genotypes could indicate they are not all descending from the same source, and it is possible that sweet potato is occasionally (re-)infected from an unknown source plant. Alternatively, the sequence variation could be a result of viruses replicating in the same host genotype for extensive periods of time. Electron microscopic studies by Sim et al. (2008) claimed to identify *Badnavirus*-like particles in *Ipomoea cordatotriloba* graft inoculated with sweet potato accession W-285 plants, and it could be interesting to survey more wild *Ipomoeas* species as possible sources of SPPV.

We conclude that the SPPV we detected correspond to episomal viruses, because (i) fragments covering the complete genomes can be amplified from plants, (ii) sequences are clearly not integrated in the genome in the tested genotype, (iii) sequences are transmitted to the progeny through seed, (iv) sequences can be serially graft transmissible to other plants, and (v) sequences show a high level of variation between plant genotypes like seen for true viruses. The extremely low titers observed suggest that they are managing to replicate in only very few cells, inducing efficient antiviral silencing in the plant (as evidenced by the high level of siRNAs found corresponding to those viruses), but are able to maintain themselves in the plant germline as evidenced by 100% transmission to offspring. Such an outcome could be imagined if viruses were specialized in survival in meristematic cells, but poorly adapted to survival in developed tissues and cells. In the future, cytochemical studies on SPPV in meristems of sweet potato plants might provide evidence for such hypothesis.

Based on their apparent universal presence in sweet potatoes and lack of obvious symptoms and vertical transmission over generations, SPPV could be considered among the persistent (or cryptic) viruses (Roossinck, 2011, 2015). Previously identified persistent viruses have been exclusively RNA viruses belonging to specific families such as Partitiviridae and Totiviridae (dsRNA) or Endornaviridae (ss + RNA). Persistent viruses are characterized by vertical transmission, from seed and/or pollen and cell-to-cell by redistribution in dividing cells. They lack movement proteins and in the case of endornaviruses even lack any discernible proteins besides the replicase (containing domains for RdRp, helicase, methyltransferase, and glycosyltransferase). Because they also lack any discernible symptoms in infected plants, they have been considered commensal or mutualistic in their interaction with plants, although mutualistic interaction has only been proven in a couple of cases (Nakatsukasa-Akune et al., 2005; Marquez et al., 2007). On the other hand, a weak parasitic interaction between bell pepper endornavirus and the host has been shown by Escalante and Valverde (2019), and a mutualistic interaction between endornaviruses and common bean has been shown by Khankhum and Valverde (2018). The sweet potato badnaviruses described in this study are unique from previously described persistent RNA viruses by their extremely low titers, the apparent lack of uninfected genotypes, the presence of a movement protein domain, and a circular DNA genome. Whether the presence of SPPV in all the genotypes we tested results from a mutualistic interaction or even a process of human selection remains to be determined but is certainly an intriguing possibility.

## Materials and Methods

### Plant Materials and Viruses

Plant materials used are summarized in Table 2. A total 78 accessions from the worldwide sweet potato collection (including five newly acquired accessions that do not yet have numbers assigned) and three related wild *Ipomoea* species available at the International Potato Center (CIP) gene bank were evaluated by PCR for presence of SPPV. Since their original acquisition, they have been maintained in an insect-proof greenhouse at 27°C ± 1°C at CIP as a backup to the *in vitro* collection. cv. “Huachano” used in this study originated from *in vitro* “virus-free” plants that had passed through thermotherapy and meristem tip culture (Kreuze et al., 2009). A mapping population of a cross between cv. “Beauregard” and “Tanzania” was described previously (Kyndt et al., 2015). Plants of the universal sweet potato virus indicator *I. setosa* and one accession of *I. tiliacea* were grown from seeds produced at CIP virology unit.

### Nucleic Acid Extractions

Total DNA from infected *Ipomoea* species leaves was extracted using the CTAB method (Khankhum and Valverde, 2018). Leaf tissue (approximately 250–400 mg) was ground to a fine power in liquid nitrogen using a mortar and pestle, in the presence of 2 mL of extraction buffer, followed by an incubation period at 60°C for 30 min and addition of an equal volume of chloroform: isoamyl alcohol (24:1). The homogenate was vigorously shaken at room temperature for 10 min using a vortex and after centrifugation at 12,000 × *g* for 10 min; the supernatant (∼500 μL) was recovered, mixed with same volume of isopropanol, and centrifuged at 12,000 × *g* for 10 min. The precipitated DNA was washed with 70% ethanol, dried, resuspended in 100 μL of nuclease-free water, and kept at −20°C until analysis.

Total RNA was extracted using a modified CTAB method (adapted from Doyle and Doyle, 1987). Fresh leaf tissue was ground with a hand roller. CTAB buffer (10 × vol/wt) was added, followed by centrifugation at maximum speed for 5 min at room temperature. Subsequently, an equal volume of chloroform:IAA (24:1) was added, and the homogenate was mixed thoroughly before centrifugation again at maximum speed for 5 min. The supernatant was carefully removed and mixed with an equal volume of 4 M LiCl and left overnight on ice in fridge. The precipitated RNA was centrifuged for 20 min at maximum speed. The pellet was washed with 70% ethanol, dried, and kept at −70°C until analysis.

### PCR Amplifications, Sequencing, and Sequence Analysis

Polymerase chain reactions were performed in a total volume of 25 μL containing 2 mM MgCl_2_, 1 × PCR buffer, 0.2 mM dNTPs, 0.2 μM of each primer, 0.02 units Taq DNA polymerase (Promega, Madison, WI, United States) and 1 μL (100 ng) of DNA sample. DNA from healthy *I. setosa* plants was included in these experiments as negative controls. PCR amplification of virus-specific fragments of SPPV-A and SPPV-B from cv. Huachano, was performed using primers designed based on previously reported partial sequences (Kreuze et al., 2009). Additional primers were designed based on the conserved functional domains present in the putative polyprotein encoded by open reading frame (ORF) 3 for detection of SPPV-A and SPPV-B in germplasm and grafting experiments (Table 1). PCR was performed in a DNA thermal cycler (Applied Biosystems, Foster City, CA, United States) with an initial denaturation cycle for 2 min at 94°C, followed by 35 cycles for 30 s at 94°C, 30 s at 56°C, 1 min at 72°C, and a final extension for 10 min at 72°C. The amplified products were loaded in a 1% agarose gel stained with GelRed^TM^ (Biotium, Hayward, CA, United States). Amplified fragments were cloned into pGEM-T Easy (Promega). Sequencing of PCR amplified fragments using the Sanger method was performed by Macrogen (Seoul, Korea)

Nucleic acid alignments and phylogenetic analysis were performed using Mega7 (Lodhi et al., 1994)^1^ using maximum likelihood and the substitution models calculated to best fit the alignment data.

### Quantitative Real-Time PCR

Sweet potato plants (three per treatment) were infected with SPFMV, SPCSV, or both viruses (SPVD) under controlled greenhouse conditions in Lima, Peru. Cuttings were taken from infected and non-infected plants and grown for 3 months after which leaves were collected from basal, middle, and top of each plant. Each leaf was considered as a separate sample. Total RNA was extracted using CTAB as described above. One microgram of total RNA was treated with 2 U of Turbo DNA-free^TM^ (Ambion, Austin, TX, United States) in a total volume of 10 μL according to the manufacturer’s protocol. After heat deactivation of the DNase enzyme, cDNA synthesis was carried out using 1 μL of the DNase-treated RNA, random primers (Invitrogen, Carlsbad, California, United States), and Superscript^TM^ III reverse transcriptase (Invitrogen) in a total volume of 20 μL according to the manufacturer’s protocol.

The qPCR primers were for actin, SPPV-A, and SPPV-B (Table 1) and were designed using the “Primer3” open source bioinformatics tool^2^. Primers for cytochrome oxidase (Cox) have been previously reported (Kumar et al., 2016).

The qPCR experiment was set up with three replicates per sample per plate. The Power SYBR^®^ Green PCR Master mix (Applied Biosystems) was employed for the qPCR with 4 μL of cDNA solution in a volume of 10 μL according to the manufacturer’s protocol. The reaction and the detection of the fluorescent signal were performed with the Mx 3005P qPCR System (Stratagene, San Diego, CA, United States). Actin and Cox genes were used as internal control and reference genes for data normalization. The data analysis was carried out using the 2^(–ΔΔ*Ct)*^ method (Livak and Schmittgen, 2001) to determine relative RNA levels. The REST2009 software (Qiagen, Hilden, Germany), which uses pairwise fixed reallocation randomization tests to infer statistical significance, was used to determine statistical significances in relative expression between different samples (Pfaffl et al., 2002).

### Southern Blots

A plasmid containing SPPV insert was used to synthesize non-radioactive probe using the PCR DIG Probe Synthesis Kit (Roche, Basel, Switzerland) with the primers SPbadnaB 5704f and SPbadnaB 6262r (Table 1), which amplified a ∼600-bp fragment of ORF 3b region. The probe was amplified with a thermal cycler (Piko, Finnzymes) using 30 cycles, each consisting of 30 s at 95°C, 30 s at 60°C, and 40 s at 72°C. A final step of 7 min at 72°C was included. Total DNA from sweet potato cv. “Huachano” foliar tissue was extracted using the CTAB method as described above. Extracted DNA (30 μg) was digested with *Eco*RI and separated by 0.8% agarose gel electrophoresis in TAE containing GelRed^TM^ overnight at 30 v. The plasmid containing the SPPV insert was linearized with *Pst*I and used as a positive control. After depurination, denaturation, and neutralization steps, DNA was transferred to a positively charged nylon membrane and fixed with ultraviolet light treatment (UV Stratalinker 2400; Stratagene). DNA was then prehybridized, hybridized, and developed with CDP-Star, ready-to-use kit (Roche) following the manufacturer’s procedures and Kodak Biomax light film (Sigma).

### Graft Transmissions

*Ipomoea setosa* and two sweet potato genotypes (Amarilla and Man Sai Deng, CIP Germplasm accession numbers 401243 and 440197, respectively), which tested negative for SPPV-A or SPPV-B by PCR screening, were selected for graft transmission experiments from cv. Huachano. Plants were tested by PCR for SPPV-A and SPPV-B using generic SPPV primers before graft inoculation, after which they were inoculated by side grafting a single node including leaf of the sweet potato cv. Huachano, which was either not infected by any other virus, or infected by SPFMV and SPCSV. All plants were maintained in a greenhouse under controlled conditions at 27°C ± 1°C and monitored for symptoms up to 8 weeks and tested by PCR at 25 days after grafting. The success of the graft union was confirmed by survival of the grafted scion throughout the experiment. PCR fragments amplified by SPPV-A– and SPPV-B–specific primers were sequenced to corroborate the results. To confirm that positive PCR results in graft inoculated *I. setosa* plants were not due to passive transmission of virus from the grafted sweet potato scion, serial transmission was performed by grafting scions from the first *I. setosa* plants to two new *I. setosa* plants. The serially grafted *I. setosa* plants were tested by PCR using the generic primers RT-F and RT-R at 21 days after inoculation.

### siRNA Sequencing and Assembly

To evaluate the effect of coinfection of SPFMV and SPCSV on SPPV siRNA levels, leaves from the middle section of vines of 1-month-old “Huachano” plants infected or not with SPFMV, SPCSV, or both viruses (SPVD) were used for RNA extraction. RNA was extracted using Trizol reagent according to the manufacturer’s instructions. RNA was run in a 3.5% agarose gel and the band corresponding to siRNAs cut and purified using quantum prep gel purification columns (Bio-Rad, Hercules, CA, United States). Purified siRNAs were sent to Fasteris Life Sciences (Plan-les-Ouates, Switzerland) for sequencing on an Illumina Hiseq 2000. Small RNA sequences were downloaded and are accessible^3^; siRNA sequences were mapped against the genomes of SPPV-A and SPPV-B using MAQ, and coverage of their respective genomes by siRNAs was visualized using a custom script (available from authors upon request).

To identify SPPV infecting sweet potato cultivars collected from the field in Africa, RNA was extracted from leaves of seven different plants. They were combined with 13 additional samples from potato and other plant species, processed, and sequenced as described above^4^. Sequences were *de novo* assembled using velvet as described previously, and contigs were submitted to BlastX at NCBI selecting badnaviruses as organism search set. The hit tables were downloaded and imported into Microsoft Excel (Redmond, WA, United States) for presentation (Supplementary Data S1), and contigs with hits were aligned to SPPV-A and SPPV-B sequenced for design of degenerate primers able to identify all SPPV variants.

## Data Availability Statement

The datasets generated for this study can be found in the https://research.cip.cgiar.org/confluence/display/cpx/CIP.sweetpotato.2014.

## Author Contributions

JK made the experimental design and wrote the draft manuscript. AP, MG, and WC carried out the laboratory experiments. AP and MG analyzed the data. All authors interpreted the data and contributed with the revision and analysis of the data.

## Conflict of Interest

The authors declare that the research was conducted in the absence of any commercial or financial relationships that could be construed as a potential conflict of interest.

## Footnotes

1 www.megasoftware.net

2 http://primer3.sourceforge.net/

3 https://research.cip.cgiar.org/confluence/display/cpx/CIP.sweetpotato.2014

4 https://research.cip.cgiar.org/confluence/display/cpx/GAF13-14
